# Effects of Different LED Spectra on the Antioxidant Capacity and Nitrogen Metabolism of Chinese Cabbage (*Brassica rapa* L. ssp. *Pekinensis*)

**DOI:** 10.3390/plants13212958

**Published:** 2024-10-23

**Authors:** Jie Li, Yubing Liu, Junwei Wang, Mingyue Liu, Yanling Li, Jingyuan Zheng

**Affiliations:** 1 Institute of Vegetables, Hunan Academy of Agricultural Sciences, Changsha 410125, China; licky0725@126.com (J.L.); 13787107749@163.com (Y.L.); 2 Longping Branch, College of Biology, Hunan University, Changsha 410125, China; 3 College of Horticulture, Hunan Agricultural University, Changsha 410128, China; 17835395172@163.com (Y.L.); junweiwang87@126.com (J.W.); liumingyue58@126.com (M.L.)

**Keywords:** LED light combination, growth and development, antioxidant enzyme, nitrogen metabolism

## Abstract

Light quality optimization is a cost-effective method for increasing leafy vegetable quality in plant factories. Light-emitting diodes (LEDs) that enable the precise modulation of light quality were used in this study to examine the effects of red-blue (RB), red-blue-green (RBG), red-blue-purple (RBP), and red-blue-far-red (RBF) lights on the growth, antioxidant capacity, and nitrogen metabolism of Chinese cabbage leaves, while white light served as the control (CK). Results showed that the chlorophyll, carotenoid, vitamin C, amino acid, total flavonoid, and antioxidant levels of Chinese cabbage were all significantly increased under RBP combined light treatment. Meanwhile, RBG combined light treatment significantly increased the levels of amino acids but decreased the nitrite content of Chinese cabbage. In addition, RBF combined light treatment remarkably increased the amino acid levels but decreased the antioxidant capacity of Chinese cabbage. In conclusion, the addition of purple light to red-blue light was effective in improving the nutritional value and antioxidant capacity of Chinese cabbage. This light condition can be used as a model for a supplemental lighting strategy for leafy vegetables in plant factory production.

## 1. Introduction

With the increasing demand for nutritional and bioactive vegetables, environment-controlled vegetable cultivation has been widely practiced in daily life given its safe and efficient production management. A plant factory with a hydroponic system, in which leafy green vegetables are the common crops, exhibits characteristics of high yield, high quality, stability, and no environmental pollution [1]. Environmental factors in a plant factory, such as light, temperature, and CO_2_ concentration, may be adjusted to specifically target the growth and quality of leafy green vegetables. The regulation of the light condition is the most effective and economical technique to improve vegetable quality and nutritional value [2]. A suitable light condition for plant growth and nutrient enrichment can maximize the use of light energy resources. It can be created by adjusting light quality, light intensity, and photoperiod [3,4].

Light quality plays a significant role in plant growth, metabolite biosynthesis, and morphogenesis [5,6]. Plants sense the spectra of different bands through various light receptors (e.g., phytochrome, cryptochrome, and phototropin), and they adjust their morphology, physiological function, and gene selective expression in accordance with the changes in the surrounding light condition [7]. A previous study showed that either red or blue light generally affects plant morphogenesis and the accumulation of chlorophyll, carotenoid, and soluble sugar [8,9,10]. Either red or blue light has also been reported to promote the accumulation of plant secondary metabolites [11]. Numerous studies have demonstrated that the mixture of red and blue lights exhibits better effect on plant growth and quality than either red or blue light alone [12,13,14]. Thus, the combination of red and blue lights has been frequently used as a light source in plant factories. Many studies have shown that adding other monochromatic lights based on the red-blue (RB) light combination can help improve crop quality. For example, combining red, blue, and green lights can reduce the amount of nitrate and improve nutritional values [15]. Far-red light, combined with red and blue lights, increases the vitamin C content but decreases the nitrate content of lettuce leaves [16].

Reactive oxygen species (ROS) are crucial metabolic by-products that control plant growth and development [17]. Excess ROS production causes oxidative damage to plants when they suffer from biotic and abiotic stresses [18]. With the aid of sophisticated and extremely complicated antioxidant systems, a strictly regulated balance between synthesis and breakdown determines ROS levels. A set of ROS scavenging systems, such as antioxidant enzyme systems [e.g., superoxide dismutase (SOD), peroxidase (POD), and catalase (CAT)] and bioactive substances (e.g., polyphenols and flavonoids), have been produced through the long-term evolution of plants [19]. Recent research has shown that ROS content and antioxidant activity are impacted under different light-emitting diode (LED) spectra. Moreover, ROS could be a signal or precursor substance to activate the antioxidant enzymes [20]. Edible vegetables with enhanced antioxidant capacity may serve as essential natural antioxidants for free radical scavenging against damaging oxidants in the human diet.

Nitrogen metabolism is the most important physiological metabolic process for crop growth and development. The high metabolic activity, dynamic growth, and development processes of nitrogen exert a direct effect on the synthesis of proteins, mineral nutrient uptake, and the formation and transformation of photosynthetic products. they also have significant effects on the yield and quality of vegetables [21]. Key enzymes, including nitrate reductase (NR), nitrite reductase (NiR), glutamine synthetase (GS), glutamic acid synthetase (GOGAT), and glutamate dehydrogenase (GDH), control the nitrogen metabolism process. In particular, NR is the rate-limiting enzyme of the nitrate assimilation process, which is an important stage of nitrogen metabolism in plants. Nitrogen metabolism is also influenced by light quality. After sensing a light signal, the phytochrome regulates the gene expression of enzymes related to nitrogen metabolism through the HY5 pathway, affects the activity of enzymes (NR, GS, and GOGAT), and then influences the contents of nitrate nitrogen, soluble proteins, and amino acids [22]. Leafy vegetables in hydroponic systems are generally nitrophilic. However, excessive absorption of nitrogen produces nitrite, which is harmful to the human body. Therefore, regulating the absorption and transformation of nitrogen via light quality is a convenient and fast technique used in plant factories.

Chinese cabbage (*Brassica rapa* L. ssp. *pekinensis*) originated in China and is highly preferred by various consumer groups because of its small size, bright color, crisp texture, and richness in various vitamins, minerals, and trace elements that are essential in Chinese eating habits. Chinese cabbage is a type of leafy vegetable and is highly suitable for hydroponic cultivation in plant factories. In accordance with previous research, light quality is an effective and fast method for the regulation of vegetable growth, development, and nutrition. Red-Blue (RB) light has been extensively studied in plant factory cultivation experiments. It is assumed that adding other monochromatic light sources to the same proportion of RB light does not affect the growth of Chinese cabbage but can improve its antioxidant capacity and nutritional quality. To find the appropriate combination of light, the effect of RB light, and RB light with the addition of green, purple, and far-red light on the quality, nitrogen metabolism, and antioxidant capacity of Chinese cabbage were explored in the present study. The results are not only important theoretically but also practically for optimizing light conditions in plant factories to produce high-quality Chinese cabbage.

## 2. Materials and Methods

### 2.1. Plant Material

**Figure 1:**
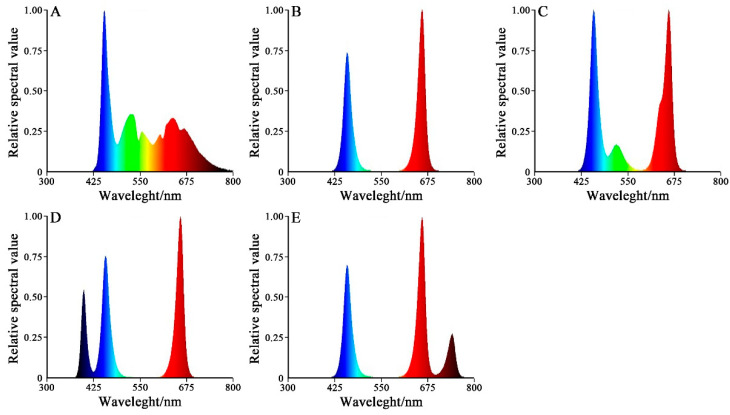
Relative spectral value of white light and four other treatments. Note: (**A**): CK: white light, (**B**): RB: 75% red + 25% blue LED, (**C**): RBG: 60% red + 20% blue + 20% green LED, (**D**): RBP: 60% red + 20% blue + 20% purple LED, (**E**): RBF: 60% red + 20% blue + 20% far-red LED.

Chinese cabbage (‘Zaoshu No. 5’ from Hangzhou Liuhe, Co., Ltd., Hangzhou, China) was used in this study. The experiment was performed in the artificial climate chamber of Hunan Agricultural University. The seeds were directly seeded into 50 -cell plastic trays. Uniform seedlings were picked at two leaves and placed in a hydroponic tank (180 mm × 650 mm × 100 mm) with a half unit of Japanese garden test nutrient solution [23,24], and the specific formula is provided in Table S1. After a week of pre-culture under white LED light with a 14 h photoperiod (150 mmol·m⁻^2^·s⁻^1^), the seedlings were randomly divided into five groups to receive different illumination treatments. The five different illumination treatments were as follows: (1) CK, i.e., white light treatment, as control; (2) RB: 75% red + 25% blue; (3) RBG: 60% red + 20% blue + 20% green; (4) RBP: 60% red + 20% blue + 20% purple; (5) RBF: 60% red + 20% blue + 20% far-red. The spectrum is shown in Figure 1. Light intensity was adjusted to 150 ± 10 mmol·m⁻^2^·s⁻^1^ for all treatments. The parameters of the artificial climate chamber were set as follows: 25 °C during the day, 18 °C at night, 60% air humidity, and a 14 h/10 h photoperiod. The nutrient solution was set as follows: electrical conductivity (EC) of 1.2 dS·m⁻^1^; pH of 6.4; and dissolved oxygen at 8.0 ± 0.2 mg·L⁻^1^, which was replaced every 7 days. The experiment used 225 seedlings across five treatments, including three repetitions per treatment (15 plants per repetition) in a completely randomized block design. The experiment was performed in three independent regions for the repetitions.

At 25 days after light treatment, five plants were chosen at random to measure plant height, stem width, fresh weight of the shoot, and dry weight of the shoot. The height from the base of the stem to the highest point of the plant was defined as plant height, which was measured with a ruler. The thickness at a distance of 1 cm from the base of the plant stem was defined as stem width and measured using a vernier caliper. The fresh weight of the shoot was weighed using a one -thousandth balance after removing the roots. The shoots of Chinese cabbage were placed in a baking oven at 65 °C until they reached a constant weight, then the dry weight of the shoot was weighed.

Ten plants under each treatment were selected, and the leaves were gathered. The samples were cleaned, blotted dry with filter paper, and then instantly frozen with liquid nitrogen. The samples were stored in an ultra-low-temperature refrigerator at −80 °C for the measurement of quality indexes, antioxidant capacity, nitrogen metabolism indexes, and gene expression levels. The remaining samples were washed with deionized water, dried with absorbent paper, and then freeze-dried to a constant weight at −20 °C before being crushed and sieved to assess the concentrations of amino acids, total phenols, and flavonoids.

### 2.2. Determination of Growth Parameters, Photosynthetic Characteristics and Fluorescence Parameters

The net photosynthetic rate (Pn) of new fully expanded leaves of Chinese cabbage was determined using a portable photosynthesis open system (model 6400; Li-COR, Lincoln, NE, USA) between 9:00 and 11:00 on a sunny day. The leaf temperature and relative humidity were maintained at 28 °C and 50%, respectively, and the light flux intensity was 600 µmol photons m⁻^1^·s⁻^1^. Data were recorded after equilibration (approximately 10 min).

Maximum quantum yield (Fv/Fm), non-photochemical quenching (NPQ), quenching parameter (qP), electron transfer rate (ETR), and OJIP-test of fully expanded leaves of Chinese cabbage were determined after the plants were kept in darkness for 15 min using a chlorophyll fluorescence analyzer (FluorPen FP 110; Czech PSI Company, Prague, Czech Republic). The various fluorescence parameters were measured after 30 min of darkness.

### 2.3. Chemical Analyses

#### 2.3.1. Measurement of Leaf Pigment Content

Colorimetric analysis was performed to determine the amount of pigment in the leaves. First, 0.2 g of fresh leaves (the third fully expanded leaf) were soaked in 10 mL of mixture of acetone, ethanol, and water (4.5:4.5:1, v/v/v) under dark conditions until they turned white. The mixture absorbance was determined at 440 nm (OD_440_), 645 nm (OD_645_), and 663 nm (OD_663_) using a microplate reader. In accordance with a previous report [25], the chlorophyll and carotenoid content were calculated as follows:

$${Chlorophyll\;a\;content\;(mg/L)=12.7\times OD}_{663}-2.69\times {OD}_{645}\;{Chlorophyll\;b\;content\;(mg/L)=22.9\times OD}_{645}-4.68\times {OD}_{663}\;{Carotenoid\;content\;(mg/L)=4.7\times OD}_{440}-0.27\times (8.02\times {OD}_{663}+20.20\times {OD}_{645})$$

#### 2.3.2. Measurement of Total Phenolics and Total Flavonoids Content

Total phenolic content was measured via the Folin–Ciocalteu test [26]. The dry sample powder (0.1 g) was mixed with 1.5 mL of 60% ethanol and extracted for 30 min at 60 °C. The mixture was centrifuged at 10,000× *g* at 25 °C for 10 min. Then, the supernatant (10 μL) was mixed with 50 μL of 10% Folin–Ciocalteu’s phenol reagent, 50 μL of 2% sodium carbonate solution, and 90 μL of distilled water. The mixture was mixed and allowed to react for 10 min at room temperature. A microplate reader was used to detect absorbance at 760 nm. The results were represented as mg·g⁻^1^ FW.

The total flavonoid content was determined using the method published by Rao et al. [27]. Dry sample powder (0.1 g) was combined with 1.0 mL of 60% ethanol and extracted for 30 min at 60 °C. The mixture was centrifuged at 10,000× *g* at 25 °C for 10 min. Then, the supernatant (60 μL) was mixed with 120 μL of 1 M potassium acetate solution, 30 μL of 10% aluminum nitrite solution, and 90 μL of 60% ethanol. The mixture was allowed to react at 37 °C for 45 min. Absorbance was measured at 470 nm with a microplate reader. The results were represented as milligrams per gram of dry weight (mg·g⁻^1^ DW).

#### 2.3.3. Measurement of Soluble Protein, Soluble Sugar, and Ascorbic Acid Contents

The soluble protein, soluble sugar, and ascorbic acid contents of Chinese cabbage leaves were determined using kits with a microplate reader.

According to the instructions of the reagent kit (Suzhou Keming Technology Co., Ltd., Suzhou, China), the BCA method was used to determine the soluble protein content. A 0.1 g sample of tissue was weighed into 1 mL of extraction solution. The sample was homogenized in an ice bath and centrifuged at 4 °C, 8000× *g* for 10 min. The supernatant was taken for testing. 1 mL of working solution was mixed with 20 µL of distilled water, 20 µL of standard solution, and 20 µL of supernatant, respectively, and kept at 60 °C for 30 min. The absorbance values A1, A2, and A3 were measured at 562 nm, and the soluble protein content was calculated according to the following formula:

$$Soluble\;protein\;content\;(mg/g)=\frac{5\times (A3-A1)}{(A2-A1)\times W}$$

Anthrone colorimetry was conducted to determine the content of soluble sugar, and absorbance was measured at 620 nm. Briefly, a 0.1 g sample was weighed into a centrifuge tube, 1 mL of distilled water was added to obtain the homogenate, and then the mixture was placed in a 95 °C water bath for 10 min. The mixture was centrifuged at 8000× *g* for 10 min for 10 min at room temperature, and the supernatant was brought to 10 mL. An appropriate amount of distilled water and supernatant were mixed with the working solution, respectively, and then placed in a water bath at 95 °C for 10 min. The absorbance values of distilled water (A1) and supernatant (A2) were read at 620 nm. The soluble sugar content was calculated according to the following formula:

$$Soluble\;sugar\;content\;(mg/g)=\frac{1.17\times (A1-A2+0.07)}{W}$$

The determination of ascorbic acid (vitamin C, Vc) was conducted according to the instructions of the reagent kit (Suzhou Keming Technology Co., Ltd., Suzhou, China). Weigh 0.1 g of the sample and add 1 mL of reagent. After ice bath homogenization, centrifuge at 8000× *g* for 20 min at 4 °C. Take the supernatant for testing. Add 100 µL of standard solution, 800 µL of reagent 2, and 100 µL of reagent three to the colorimetric dish in sequence. Mix quickly and measure at 265 nm with a spectrophotometer. Record the absorbance values A1 and A2 at 30 s and 150 s, respectively. Replace the standard solution with the supernatant. Follow the above steps to record the absorbance values A3 and A4 at 30 s and 150 s, respectively, and calculate the Vc content according to the following formula:

$$Vc\;content\;(nmol/g)=\frac{100\times (A3-A4)}{(A1-A2)\times W}$$

#### 2.3.4. Determination of Antioxidant Capacity

The antioxidant enzyme activity (SOD, POD, CAT), H_2_O_2_ content, O_2_^·−^ content, and malondialdehyde (MDA) content of Chinese cabbage leaves were determined using kits purchased from Beijing Solarbio Science (Beijing, China). All measurements were performed according to the manufacturer’s instructions.

The DPPH, ABTS, and FRAP assays were tested using the method described by Wang et al. [28]. Briefly, the sample was ground into powder after drying for the determination of DPPH. A 50 mg dried sample was weighed and mixed with 1.0 mL ethanol and extracted at 40 °C for 30 min. Then, the mixture was centrifuged at room temperature at 8000× *g* for 10 min. 10 µL of supernatant was taken and added to 190 µL of DPPH solution with a concentration of 0.4 mM. the absorbance value of the mixture at 515 nm was measured after reacting in the dark for 10 min. For ABTS, the previous steps were the same as for DPPH, but after centrifugation, 10 µL of supernatant was added to 170 µL of ABTS+ solution. The absorbance value of the mixture was measured at 405 nm after reacting in the dark for 6 min. The FRAP assay was performed according to Benzie and Strain [29]. A 0.1 g fresh sample was weighed and extracted with 1.0 mL of distilled water. After centrifugation at 4 °C for 10 min, 6 µL of supernatant was taken and added to a mixture containing 18 µL of distilled water and 180 µL of Fe^3+^-TPTZ reagent. The absorbance value was measured at 593 nm after reacting for 10 min in darkness.

#### 2.3.5. Measurement of Nitrate, Ammonium, Nitrite, and Free Amino Acids Content

Fresh samples collected from the third-young, fully expanded leaves were used to determine the nitrate, nitrite, and ammonium content. The determination of nitrate content was performed according to the instructions of the reagent kit (Suzhou Keming Technology Co., Ltd., Suzhou, China). The principle was that nitrate reacts with salicylic acid to produce nitrosalicylic acid. A 0.1 g fresh sample was added into 1 mL of distilled water, then homogenized and placed in a 90 °C water bath for 30 min, shaking continuously. The mixture was then centrifuged at room temperature for 15 min at 12,000× *g*, and the supernatant was mixed with the working solution to test. The absorbance value of the blank control and supernatant was measured by a spectrophotometer at 410 nm, and the data were recorded as A1 and A2, respectively. The formula for calculating nitrate nitrogen content was as follows:

$$Nitrate\;content\;(mg/kg)=\frac{64.1\times (A2-A1-0.0073)}{W}$$

The detection of ammonium nitrogen content was performed according to the instructions of the reagent kit (Suzhou Keming Technology Co., Ltd., Suzhou, China). A 0.1 g sample was weighed and added into 1 mL of extraction solution, then homogenized by an organizational disruptor. The mixture was centrifuged at room temperature for 10 min at 12,000× *g*, and an appropriate amount of supernatant was taken to mix with the working solution to test the absorbance values at 580 nm by ultraviolet spectrophotometer. The value of the blank control and supernatant was marked as A1 and A2, respectively. The ammonium nitrogen content was calculated using the following formula:

$$Ammonium\;content\;(µg/g)=\frac{32.9\times (A2-A1+0.0279)}{W}$$

Nitrite content was determined according to the reagent kit instructions (Suzhou Keming Technology Co., Ltd., Suzhou, China). The principle is that ammonium reacts with ninhydrin to produce a blue compound. Briefly, a 0.2 g sample was weighed and crushed in a 2 mL centrifuge tube, then three different extraction solutions were added in turn, and finally 1 mg of powder was added. The mixture was held at room temperature for 30 min, then centrifuged at 8000× *g* for 15 min. The supernatant was taken for testing the absorbance values at 540 nm, and the value was recorded as A2, while the blank control was A1. The nitrite content was calculated as follows:

$$Nitrite\;content\;(µg/g)=\frac{4.27\times (A2-A1-0.0002)}{W}$$

Free amino acids were assayed in accordance with the method described by Shu et al. [30]. First, 0.1 g of dry samples were extracted with HCl at 110 °C for 24 h. Then, the chilled hydrolysate was filtered, dissolved, and evaporated under vacuum. The resulting dry matter was dissolved in citrate buffer (67 mM, pH 2.2) and analyzed. An automated amino acid analyzer (Hitachi L-8900, Hitachi Company, Tokyo, Japan) was used to determine the level of free amino acids.

#### 2.3.6. Measurement of Enzymatic Activity of Nitrogen Metabolism

Fresh leaf samples were used to assay NR, NiR, GS, GOGAT, and GDH activities via a colorimetric method in accordance with kit instructions (ZCiBio Co., Ltd., Shanghai, China). According to the instructions, 0.1 g of the sample was weighed and placed into appropriate extraction solutions. After centrifugation, the supernatant was taken to mix with the working solution provided in each reagent kit. A Spectrophotometer was used to detect the absorbance values at different wavelengths. Among them, NR was at 520 nm, NiR and GS were at 540 nm, and GOGAT and GDH were at 340 nm. the activity of each enzyme was then calculated according to the formula provided in the kit instructions based on fresh weight and absorbance. The amount of catalyzed production of 1 µmol NO^2−^ per gram of fresh sample per hour was regarded as one NR activity unit. The unit of NiR activity was defined as the molar mass of NO^2−^ reduced per gram of leaf per minute. The GS enzyme activity unit was defined as a 0.01 change in absorbance at 540 nm per minute per gram of tissue in each mL of reaction system. The GOGAT enzyme activity unit was defined as the consumption of 1 nmol NADH per minute per g of tissue. The definition of the GDH enzyme activity unit was the same as GOGAT.

### 2.4. RNA Extraction and Genes Expression Analysis

As described in our previous study [25], total RNA was extracted from fresh Chinese cabbage (0.1 g) using the RNA prep Pure Plant Kit: Polysaccharides and Polyphenolics-rich (Tiangen, Beijing, China) in accordance with the manufacturer’s instructions. The RNA concentration was measured and confirmed to check whether the RNA had been degraded by gel electrophoresis. cDNA was synthesized from 1 µg of total RNA using FastKing gDNA Dispelling RT SuperMix (Tiangen Biochemical Technology Co., Ltd, Beijing, China) in accordance with the manufacturer’s instructions.

For the fluorescent quantitative PCR analysis of gene expression, the sequences of *BrNR*, *BrNiR*, *BrGS*, *BrGOGAT*, and *BrGDH* were amplified with primers designed on the basis of the sequences obtained from NCBI (Supplementary Table S2). The expression data were analyzed with the 2^−ΔΔCt^ method as previously described (BioRad Real-time PCR Application guide) [31].

### 2.5. Statistical Analysis

Excel 2017 and GraphPad Prism 9 were used to process and graph the experimental data. SPSS 20.0 was used for the single-factor ANOVA, and Duncan’s test was used for multiple comparisons of significant differences (*p* < 0.05).

## 3. Results

### 3.1. Effects of Different Light Quality Combinations on the Growth of Chinese Cabbage

The plant height, stem width, fresh weight, and dry weight of the above ground portion of Chinese cabbage under different light quality combinations were measured (Table 1). Compared with CK, all the values were significantly increased under RB and RBP conditions, but interestingly, there was no significant difference between RB and RBP treatments. RBG and RBF significantly increased the fresh weight and dry weight of the shoot. In addition, RBF also showed a positive effect on plant height of Chinese cabbage.

### 3.2. Effects of Different Light Qualities on the Photosynthesis and Fluorescence of Chinese Cabbage

**Figure 2:**
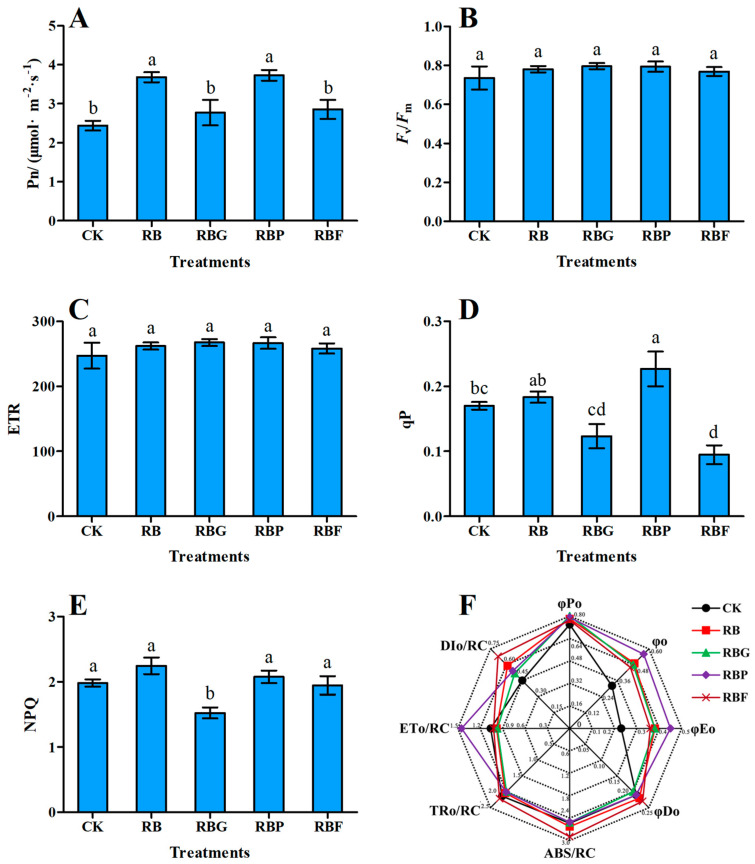
Effects of spectra on photosynthetic rate and chlorophyll fluorescence of Chinese cabbage leaves under different LED treatments (*p* < 0.05). (**A**): Net photosynthetic rate; (**B**): Maximum quantum yield; (**C**): Photosynthetic electron transfer efficiency; (**D**): Photochemical quenching; (**E**): Non-photochemical quenching; (**F**): OJIP-test parameter. Data represent means ± s.d. (n = 3 biological replicates). Significant differences were determined by Duncan’s multiple range test. The letters represent the significant difference of different treatments (*p* < 0.05).

Compared with CK, the Pn values of Chinese cabbage under other treatments were increased to varying degrees (Figure 2A). RB and RBP treatments significantly increased the Pn value by 50.80% and 52.75%, respectively, while RBG and RBF treatments did not reach a significant difference level (*p* < 0.05). The values of Fv/Fm and ETR under the other four LED lights showed no significant differences to CK (Figure 2B,C). The qP value of Chinese cabbage leaves under RBP light was significantly increased by 33.53% (*p* < 0.05), and significantly decreased by 44.12% (*p* < 0.05) under RBF lights compared to CK (Figure 2D). RBG-treated Chinese cabbage leaves showed the lowest NPQ value. The other treatments (RB, RBP, and RBF) did not significantly affect this value compared to CK (Figure 2E). OJIP-test analysis showed that RB light significantly increased the values of φo and φEo compared with white light (CK). Compared with RB light, addition of green, purple, or far-red light had no significant effect on values of φo, φEo, φPo, φDo, ABS/RC, TRo/RC, ETo/RC, and DIo/RC (Figure 2F).

### 3.3. Effects of Different Light Qualities on the Nutritional Quality of Chinese Cabbage

**Figure 3:**
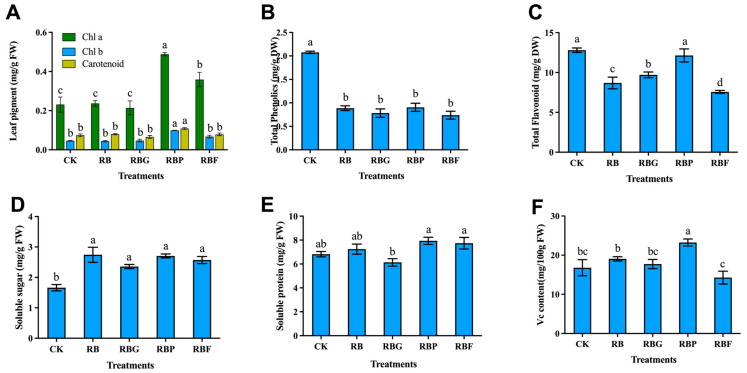
Effects of spectra on soluble sugar, soluble protein and vitamin C contents of Chinese cabbage leaves under different LED treatments (*p* < 0.05). (**A**): Content of chlorophyll a, chlorophyll b, and carotenoid; Content of: (**B**) total phenolic; (**C**) total flavonoid; (**D**) soluble sugar; (**E**) soluble protein; (**F**) vitamin C. Data represent means ± s.d. (n = 3 biological replicates). Significant differences were determined by Duncan’s multiple range test. The letters represent the significant difference of different treatments (*p* < 0.05).

The contents of chlorophyll, total phenolic, total flavonoid, Vc, soluble sugar, and soluble protein were measured (Figure 3). Compared with CK, the contents of chlorophyll a, chlorophyll b, and carotenoid in Chinese cabbage leaves under RB light were not significantly changed (Figure 3A). Compared with RB light, the content of chlorophyll a under RBP and RBF treatments was significantly higher, with RBP treatment increasing by 106.3%. The chlorophyll b and carotenoid contents under RBP treatment were significantly increased by 126.7% and 36.8%, respectively, compared to the RB treatment.

Compared with CK, the application of RB light in Chinese cabbage cultivation contributed to a decrease in total phenolics content. The addition of both green, purple, and far-red light to RB did not increase the polyphenol content of cabbage (Figure 3B). The total flavonoid content under RB, RBG, and RBF treatments was significantly decreased compared with CK, but it showed no significant difference between RBP and CK (Figure 3C). RBG and RBP treatments remarkably increased total flavonoid content by 9.8% and 39.7%, respectively, compared with RB treatment, while RBF treatment caused a 12.9% decrease in flavonoid content compared to RB treatment.

The soluble sugar content was significantly lowest in the control samples, but there were no significant differences between samples treated with RB and RB with the addition of green, purple, and far-red light (Figure 3D). The contents of soluble protein under RB, RBG, RBP, and RBF treatments did not reach a significant difference compared with CK (Figure 3E). There was no significant difference in the Vc content between CK and other treatments except RBP, which significantly increased by 38.10% compared with CK (Figure 3F).

### 3.4. Effect of Different Light Quality Combinations on the Antioxidant Capacity of Chinese Cabbage

**Figure 4:**
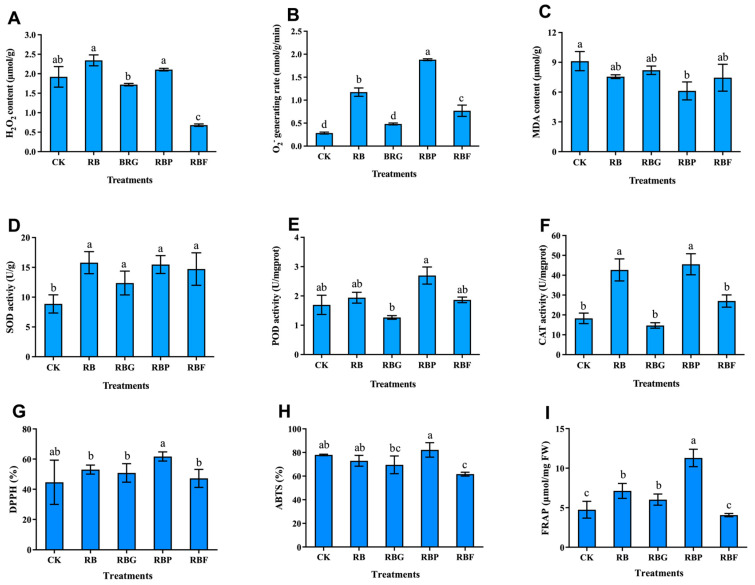
Effects of spectra on antioxidant characteristics of Chinese cabbage leaves under different LED treatments (*p* < 0.05). (**A**): content of hydrogen peroxide; (**B**): Superoxide anion generating rate; (**C**): Malondialdehyde (MDA); (**D**–**F**): the activity of superoxide dismutase (SOD), peroxidase (POD), and catalase (CAT) under different treatments; (**G**,**H**): Antioxidant evaluation indicators of Chinese cabbage under different treatments; (**I**): Ferric ion reducing antioxidant power (FRAP) under different treatments. Data represent means ± s.d. (n = 3 biological replicates). Significant differences were determined by Duncan’s multiple range test. The letters represent the significant difference of different treatments (*p* < 0.05).

The H_2_O_2_ contents and O_2_^·−^ generating rate in leaves of Chinese cabbage treated with different light were determined (Figure 4A,B). Compared with CK, the H_2_O_2_ content exhibited no significant changes under RB, RBG, and RBP treatment, but it significantly decreased by 64.58% under RBF treatment. The O_2_^·−^ generating rate under RB, RBP, and RBF treatments was significantly increased by 413.00%, 464.29%, and 253.57% compared to CK, respectively. The effect of different light quality on MDA content was relatively small, which was only significantly reduced by 32.79% under RBP treatment compared to CK (Figure 4C). SOD activity was significantly higher under all four light treatments (RB, RBG, RBP, and RBF) than CK, and the difference between the four light treatments did not reach a significant level (Figure 4D). Compared with CK, RBP treatment significantly increased POD and CAT activity by 58.82% and 149.06%, respectively, while RB treatment significantly increased CAT activity by 133.51% (Figure 4E,F).

The results showed that the addition of green, purple, or far-red light to RB treatment exhibited different effects on the antioxidant capacity of Chinese cabbage leaves (Figure 4G–I). The DPPH demonstrated an increasing trend under the four treatments compared with CK, but it showed no significant difference between them. The ABTS under RBF treatment was significantly lower than that of CK, while there was no significant difference among RB, RBG, RBP, and CK. FRAP content increased the most in Chinese cabbage treated with RBP, and the increase also occurred in RB and RBG irradiated Chinese cabbage.

### 3.5. Effect of Different Light Quality Combinations on the Nitrogen Content of Chinese Cabbage

**Figure 5:**
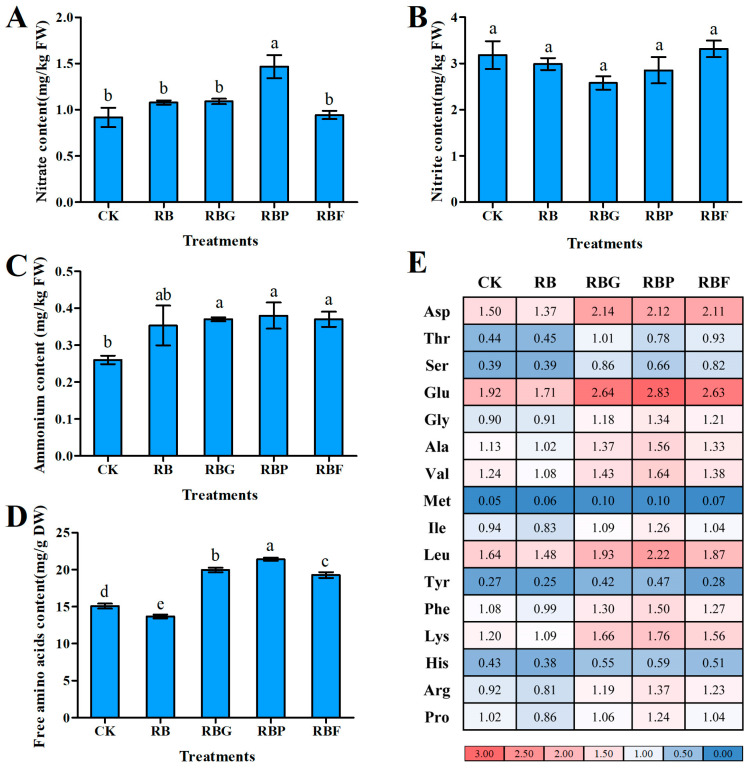
Effects of spectra on nitrate, nitrite, ammonium, and amino acids contents of Chinese cabbage leaves under different LED treatments (*p* < 0.05). (**A**–**C**): The content of nitrate (**A**), nitrite (**B**), and Ammonium (**C**); (**D**): the content of total free amino acids (sum of 16 kinds of amino acids); (**E**): The content of 16 amino acids under different treatments (The color from blue to red indicates an increase in the value). Data represent means ± s.d. (n = 3 biological replicates). Significant differences were determined by Duncan’s multiple range test. The letters represent the significant difference of different treatments (*p* < 0.05).

Compared with CK, the content of nitrate and nitrite under RB, RBG, and RBF treatments showed no significant difference, but the nitrate content of Chinese cabbage treated with RBP was significantly higher by 60.44% (Figure 5A,B). The ammonium content of Chinese cabbage leaves under RBG, RBP, and RBF treatments was significantly increased by 42.31%, 46.15%, and 42.31%, respectively, compared with CK, but they did not reach a significant value of *p* < 0.05 compared with RB (Figure 5C).

The 16 kinds of amino acids were analyzed using an amino acid analyzer, and the results are shown in Figure 5D,E. The total amino acid content (sum of 16 kinds of amino acids) under RB treatment was significantly decreased compared with CK, but it was significantly increased by 32.33%, 41.88%, and 27.83%, respectively, under RBG, RBP, and RBF lights compared to CK (Figure 5D). Overall, aspartic acid (Asp), glutamic acid (Glu), leucine (Leu), and lysine (Lys) contents were relatively higher in Chinese cabbage leaves than other kinds of amino acids (Figure 5E). These four amino acids comprise more than 40% of the total number of free amino acids found. Methionine (Met) was present in the lowest concentration among all the amino acids, accounting for only around 1% of the total. Among the 16 kinds of amino acids, except for aspartic acid and threonine, all the others achieved maximum values in RBP treatment. The content of individual amino acids under RB treatment had lower values than under RBG, RBP, and RBF treatments, particularly under RBP treatment.

### 3.6. Effect of Different Light Quality Combinations on the Nitrogen Metabolism Enzyme of Chinese Cabbage

**Figure 6:**
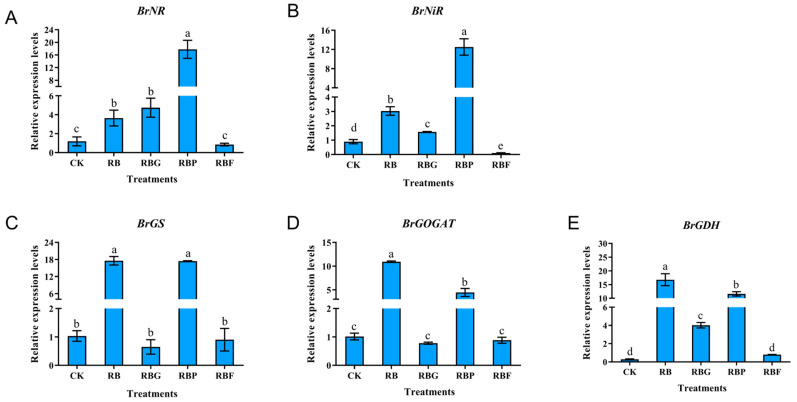
Effects of spectra on gene expression related in nitrogen metabolism of Chinese cabbage leaves under different LED treatments. The expression level of: (**A**) *BrNR*; (**B**) *BrNiR*; (**C**): *BrGS*; (**D**): *BrGOGAT*; (**E**): *BrGDH*. Data represent means ± s.d. (n = 3 biological replicates). Significant differences were determined by Duncan’s multiple range test. The letters represent the significant difference of different treatments (*p* < 0.05).

**Figure 7:**
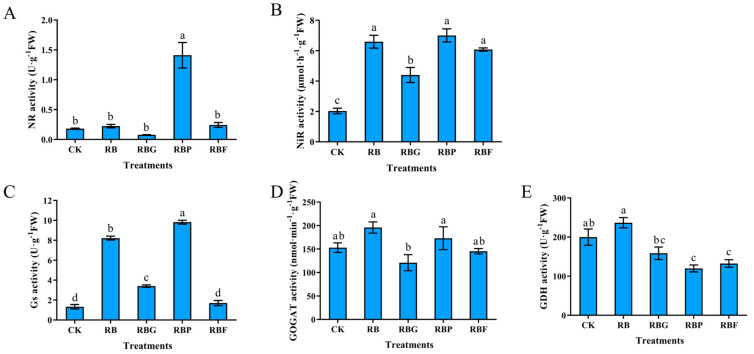
Effects of spectra on enzyme activity of nitrogen metabolism of Chinese cabbage leaves under different LED treatments. The activity of: (**A**) NR; (**B**) NiR; (**C**): GS; (**D**): GOGAT; (**E**): GDH. Data represent means ± s.d. (n = 3 biological replicates). Significant differences were determined by Duncan’s multiple range test. The letters represent the significant difference of different treatments (*p* < 0.05).

The effects of different light quality combinations on gene expression levels and enzyme activity of nitrogen metabolism in Chinese cabbage leaves are shown in Figure 6 and Figure 7. The expression levels of nitrogen metabolism enzyme genes under RB and RBP treatments were significantly higher than other treatments. Both the expression levels of *BrNR* and *BrNiR* were the highest under RBP light (Figure 6A,B). *BrNR* and *BrNiR* were also up-regulated under RB and RBG treatments but down-regulated under RBF treatment. The expression level of *BrGS, BrGOGAT,* and *BrGDH* were the highest under RB treatment, subsequently followed by RBP, which was significantly higher than CK (Figure 6C–E). However, the *BrGS* and *BrGOGAT* expression levels under RBG and RBF treatments showed no significant difference with CK. *BrGDH* expression level under RBG was significantly higher than CK, while no significant difference was shown between RBF and CK.

Compared with CK, RBP treatment significantly increased NR activity by 683.33%, while there was no significant difference under other treatments (Figure 7A). NiR activity was the lowest in control samples. Application of RB light significantly increased this value in Chinese cabbage leaves. The addition of purple and far-red light to RB did not significantly affect the NiR value, while the addition of green light contributed to a decrease in the NiR value compared with RB (Figure 7B). The highest increase in Gs activity was found for RBP-treated cabbage, slightly lower for RB, and the lowest for RBG. RBF illumination did not increase GS activity (Figure 7C). The effect of the four combination light treatments on GOGAT activity was not significant compared with CK (Figure 7D). The GDH activity under RB and RBG treatment showed no significant difference compared with CK, but it significantly decreased by 40.12% and 33.71% under RBP and RBF treatments, respectively (Figure 7E).

### 3.7. Correlation Analysis and Comprehensive Evaluation

**Figure 8:**
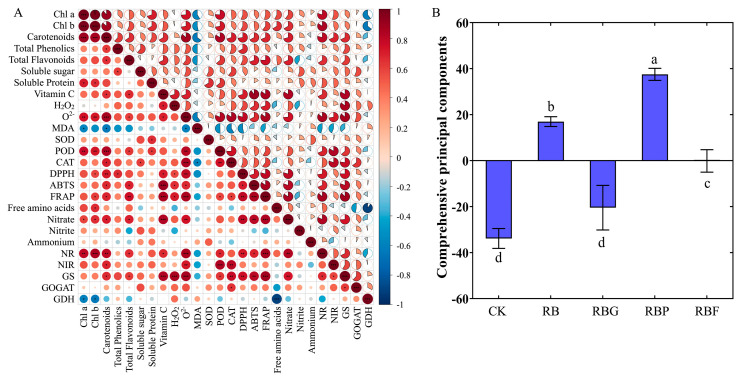
Correlation analysis and comprehensive evaluation on the growth, quality, antioxidant capacity and nitrogen metabolism of Chinese cabbage. (**A**): The correlation analysis of the indicators measured in this experiment; (**B**): Comprehensive evaluation of different light treatment on the growth, quality, antioxidant capacity and nitrogen metabolism. The asterisks in (**A**) and letters in (**B**) represent the significant difference of different treatments (*p* < 0.05).

The correlation analysis was conducted on 26 indicators of Chinese cabbage under five treatments (Figure 8). As shown in Figure 8A, there was a significant positive correlation between most indicators. The content of Chl a, Chl b, and carotenoids related to both antioxidant capacity and nitrogen metabolism processes. Vc had a positive correlation with antioxidant capacity index, especially with ROS levels (the content of H_2_O_2_, O_2_^·−^ generating rate) and DPPH, ABTS and FRAP, which showed significant positive correlation. The O_2_^·−^ generating rate was significantly positively correlated with the antioxidant enzyme system and significantly correlated with nitrogen assimilation processes, but negatively correlated with MDA content. The content of nitrate was significantly positively correlated with Vc, O_2_^·−^ generating rate, Gs, and NR activity. Interestingly, the GDH activity showed a negative correlation with most other indicators, especially free amino acides.

According to the measurement parameters under different treatments, the comprehensive evaluation of different LED treatments on Chinese cabbage was calculated (Figure 8B). The results showed that RBP treatment had a significantly positive effect on the antioxidant capacity and nitrogen metabolism of Chinese cabbage, followed by RB. Compared with RB and RBP, treatments of RBF, RBG, and CK got a negative score, though RBF and RBG showed no significant difference compared to RB.

## 4. Discussion

### 4.1. Combined Light Quality Had Positive Effect on the Chlorophyll Content and Photosynthesis of Chinese Cabbage

Photosynthesis is the foundation of plant growth and development. Light quality affects the opening and closing of stomata and also has an effect on the light absorption characteristics of photosynthetic pigments in plants. Red and blue lights are frequently used as illumination for hydroponic vegetables in plant factories due to their optimal light spectra for plant photosynthesis [32,33]. Red light is beneficial for the accumulation of photosynthetic products, while blue light promotes the development of chloroplasts. Therefore, RB light can enhance plant photosynthesis (Figure 2A). The presence of purple or far-red light stimulates chlorophyll biosynthesis [25], which is assumed to be a consequence of cryptochrome and phytochrome working together [34]. This notion is consistent with our finding (Figure 3A). A higher chlorophyll or carotenoid content, which can be related to higher photosynthesis activity [35], may explain the high Pn value under RBP treatment, which showed the highest content of chlorophyll and carotenoid (Figure 2A and Figure 3A). However, the Pn value under RBF treatment was lower than that under RB light, although the chlorophyll a content under RBF was higher. That may be because of a certain competition or imbalance between far -red light and red light in activating the plant’s light system. The specific mechanism needs further research. The Pn value of Chinese cabbage showed a decrease under RBG treatment compared to RB treatment, and the plant height, stem width, and shoot FW also significantly decreased, suggesting that the addition of green light exerted negative effects on the photosynthesis and growth of Chinese cabbage. A similar result was reported by a previous study [25].

### 4.2. Effect of Combined Light Quality on Antioxidant Capacity and Quality of Chinese Cabbage

ROS levels in plants are determined by a finely controlled balance of synthesis and breakdown accomplished by sophisticated and complicated antioxidant systems [36]. When plants are subjected to adverse stress, ROS are produced during basic metabolism in many subcellular compartments, including photosynthesis in chloroplasts. The O_2_^·−^ at the PSI system reducing side is reduced by the electrons received from the PSII system. When NADP+ is insufficient, excess O_2_^·−^ is produced and continuously converted into other ROS forms, such as H_2_O_2_ and OH^−^ [17]. Excessive ROS can cause damage to plants. So, to avoid harm, the ROS level in plants is maintained by antioxidant systems, such as the antioxidant enzyme system, Vc, total phenols, and flavonoids [37,38]. In our study, Chinese Cabbage treated with RB exhibited higher O_2_^·−^ generating rate than CK (Figure 4B), which induced a stress response in plants. As a result, higher levels of SOD activity and CAT activity were observed under RB compared to CK (Figure 4D,F). Short-wavelength light, such as blue, purple, or ultraviolet, has higher energy at the same photon flux density than long-wavelength light, such as red or far-red light. Plant antioxidant activity is stimulated in response to short-wavelength light due to the increase in photo -oxidative strain [39,40]. Consequently, the addition of purple light caused a higher O_2_^·−^ generation rate in Chinese cabbage than RB, and induced the increase in POD activity, total flavonoid content, and Vc content, which are beneficial for human health. In addition, lower ROS levels (H_2_O_2_ content and O_2_^·−^ generation rate) were observed under RBG and RBF treatments compared with RB (Figure 4A,B). The photon flux density of red and blue lights under RBG and RBF lights was only 80% of that in RB treatment in the present investigation. This result could be explained by higher optoelectronic production under RB light than under RBG and RBF lights due to the more effective red and blue lights. In plants, ROS can act as signal substances in active antioxidant systems for maintaining ROS balance. The lower antioxidant enzyme activities observed under RBG and RBF may result from the lower ROS content caused by the addition of green or far-red light.

### 4.3. Adding Purple Light Is Beneficial to Nitrogen Metabolism

Nitrogen metabolism can be divided into nitrate reduction and ammonium assimilation. NR mostly exists in the cytoplasm and catalyzes the reduction of nitrate into nitrite. GS reacts with GOGAT to produce amino acids. The activity of NR can reflect the nitrogen use efficiency of plants, while the activities of GS and GOGAT can reflect the intensity of the nitrogen assimilation ability of plants. As the direct product of ammonium assimilation, amino acids are not only the primary raw materials for protein synthesis but also the major source of protein degradation products. Moreover, the metabolism of amino acids in green vegetables can contribute some essential amino acids to humans, reflecting one aspect of vegetable nutritional value. For green leafy vegetables in a hydroponics system, nitrogen metabolism is exuberant in the leaves due to adequate nitrogen nutrients [41]. A number of studies have revealed that light plays a critical role in regulating nitrogen metabolism [13,42]. In the present study, NiR activity was induced higher by RB treatment compared with CK (Figure 7B), which can be attributed to the up-regulation of *BrNiR* (Figure 6B). However, the activity of GOGAT and GDH under RB showed no significant difference compared with CK, though the expression levels of *BrGOGAT* and *BrGDH* were significantly higher. feedback regulation of proteins synthesized by gene regulation during nitrogen assimilation, resulting in unchanged enzyme activity. However, the specific mechanism needs further research. Compared with RB, NR activity was increased by adding purple light (Figure 7A), which can benefit from higher nitrate content (Figure 5A). This result suggests that purple light can promote NO_3_^−^ absorption [38].

In addition, RBP or RBF treatments exhibited a positive effect on the accumulation of amino acids and proteins (Figure 5D and Figure 3E). Under RBP conditions, amino acid accumulation may benefit from the increased GS activity (Figure 7C), but analysis of the mechanism of lower nitrogen metabolism enzyme activity indicated that further research is required for the RBF treatment. Green light addition resulted in decreased enzyme activity (GS, GOGAT, and GDH) (Figure 7C–E) and increased amino acid content (Figure 5D), which is consistent with the findings of earlier research [43,44]. The addition of green light was hypothesized to prevent the production of protein precursors, causing a buildup of amino acids. However, the suppression of enzyme activity induced by higher amino acid content led to feedback control of nitrogen absorption, nitrate reduction, and ammonium assimilation (NR and NiR) (Figure 7A,B), which requires further study.

## 5. Conclusions

LED spectra showed substantial effects on the pigment, quality, nitrogen metabolism, and antioxidant capability of Chinese cabbage. The results of our study showed that the addition of purple light to red-blue light increased the Pn of Chinese cabbage, thus resulting in higher biomass. The combination of RBP light can lead to an increase in ROS levels in Chinese cabbage, thereby increasing the content of antioxidant substances (such as total flavonoids and Vc). In addition, RBP treatment also increased the content of amino acids and soluble proteins by promoting the uptake and accumulation of nitrogen nutrition. In accordance with the results, RBP light can increase the nutritional value of Chinese cabbage. This study can be used as a reference in supplemental lighting strategies for leaf vegetables in plant factory production.

## Figures and Tables

**Table 1:** Effects of spectra on the growth of Chinese cabbage under different LED treatments.

Treatment	Plant Height (cm)	Stem Diameter (mm)	Shoot Fresh Weight (g·plant^−1^)	Shoot Dry Weight (g·plant^−1^)
CK	26.53 ± 0.26 b	5.15 ± 0.11 b	59.37 ± 0.79 c	2.57 ± 0.10 d
RB	28.37 ± 0.32 a	6.89 ± 0.17 a	100.67 ± 1.13 a	4.39 ± 0.09 ab
RBG	26.67 ± 0.23 b	5.68 ± 0.28 b	80.03 ± 1.81 b	3.92 ± 0.30 b
RBP	28.60 ± 0.17 a	6.51 ± 0.17 a	99.67 ± 3.29 a	4.53 ± 0.15 a
RBF	29.23 ± 0.20 a	5.48 ± 0.18 b	82.13 ± 4.28 b	3.18 ± 0.07 c

CK: white LED light, RB: 75% red + 25% blue LED, RBG: 60% red + 20% blue + 20% green LED, RBP: 60% red + 20% blue + 20% purple LED, RBF: 60% red + 20% blue + 20% far-red LED. The letters represent the significant difference of different treatments (*p* < 0.05).

## Data Availability

The original contributions presented in the study are included in the article/Supplementary Material, further inquiries can be directed to the corresponding author.

## Supplementary Materials

The following supporting information can be downloaded at: https://www.mdpi.com/article/10.3390/plants13212958/s1, Table S1: Formula of Japanese Garden Test Nutrient Solution (1.0 unit); Table S2: List of primers used for RT-qPCR in Chinese cabbage.
