# Supplementary material for: Effects of Different LED Spectra on the Antioxidant Capacity and Nitrogen Metabolism of Chinese Cabbage (Brassica rapa L. ssp. Pekinensis)
Source: Plants (Basel). 2024 Oct 23;13(21):2958. doi: 10.3390/plants13212958 (PMC11548317; doi:10.3390/plants13212958)
Supplement: Supplementary file 1 [file plants-13-02958-s001.zip › plants-3208237-SI.pdf]

Table S1 Formula of Japanese Garden Test Nutrient Solution (1.0 unit)

Formula composition	Macroelement				Microelement					
	$MgSO_4 \cdot 7H_2O$	$(NH_4)_2HPO_4$	$Ca(NO_3)_2 \cdot 4H_2O$	KNO_3	Fe	B	Mn	Zn	Cu	Mo
Content	492.00	152.00	944.00	808.00	3.00	0.50	0.50	0.05	0.02	0.01

Note: The content in the table is one unit of nutrient solution, and the amount used in our experiment was a half unit.

Table S2 List of primers used for RT-qPCR in Chinese cabbage

Primer Name	Sequence (5' to 3')-F	Sequence (5' to 3')-R	Gene ID
Actin	GTCCTGTTCCAGCCTTCGTTC	CAAGTCCTTCCTGATATCCACGTC	BrA06g023770.3C
NR	ATCCAAGATTCCCCAACGG	GCCTCGGTGATAAACCCTGT	BrA07g005240.3C
NiR	TCCGCCATGACTTCCTCTT	ACTCCGTAACACGAACACCTT	BrA09g010360.3C
GOGAT	TCCGAACTCTAACCGAAAT	TTGGACAACGCAATCAGC	BrA03g014440.3C
GS	CGGGTGAACCAATCCCTACG	CGACCTGGAACTCCCACTGAC	BrA02g016970.3C
GDH	CAGAACATGTGTCGGCTTGT	AAGCACTCCAAGAAGTAAATGT	BrA10g021930.3C
